# COVID-19 Myth Busters: Comparing knowledge and perceptions amongst the dental workforce at an institution in the Eastern Province, Saudi Arabia

**DOI:** 10.1371/journal.pone.0260698

**Published:** 2021-12-22

**Authors:** Shazia Sadaf, Doaa AlEraky, Faraz Farooqi, Faiyaz Syed, Muhanad Alhareky, Jehan AlHumaid

**Affiliations:** 1 Department of Dental Education, College of Dentistry, Imam Abdulrahman Bin Faisal University, Dammam, Saudi Arabia; 2 Department of Biomedical Dental Sciences, College of Dentistry, Imam Abdulrahman Bin Faisal University, Dammam, Saudi Arabia; 3 Department of Preventative Dental Science, College of Dentistry, Imam Abdulrahman Bin Faisal University, Dammam, Saudi Arabia; Mayo Clinic Minnesota, UNITED STATES

## Abstract

**Background:**

Currently, world is suffering from a respiratory disease names as COVID-19. This is a novel coronavirus (n-CoV), a new strain which has not been previously identified in humans and it has spread in more than 100 locations internationally due to which it is termed as “public health emergency of international concern” (PHEIC) by the World Health Organization So far, no study done as yet to assess whether the dental workforce is aware about the facts and myths related to Covid-19 awareness.

**Objective:**

This study aims to analyze and compare the level of awareness about the facts and myths related to COVID-19 amongst faculty, dental students and prep year students of the College of Dentistry (COD) as part of an awareness campaign.

**Methods:**

An awareness test about COVID-19 was designed using information from the World Health Organization’s (WHO) Myth Busters Awareness webpage. The questionnaire was administrated online to faculty and students, of the College of Dentistry and preparatory year students who had applied for the admission to the dental college using a secure enterprise online assessment platform (Blackboard). The tests were administered over a period of three months from March to June 2020. A written informed consent was obtained.

**Results:**

The online COVID-19 awareness test was administered to 810 participants, out of which 325 (40%) were prep year students, 429(53%%) were dental students, and 56 (7%) were faculty members. Analysis of the results showed that 86% of the Faculty were able to correctly identify the facts and the myths related to COVID-19 followed by 81% of the prep year students and 74% of the dental students. Preparatory year student’s knowledge related to COVID-19 was found to be high when compared to dental students (26.47±4.27, 23.67±6.2). Student to faculty knowledge score did not differ significantly (p = 0.808).

**Conclusion:**

This study reports about a successful pilot test conducted to assess the perceived knowledge about facts and myths related to corona virus amongst the dental workforce.

## Introduction

The recent outbreak of Coronaviruses (CoV) named as COVID-19 in more than 100 locations internationally has been termed a “public health emergency of international concern” (PHEIC) by the World Health Organization [1].

This is a novel coronavirus (n-CoV), a new strain which has not been previously identified in humans. This virus belongs to the same family of corona viruses identified in the Middle East Respiratory Syndrome (MERS-CoV) and Severe Acute Respiratory Syndrome (SARS-CoV) [1].

Coronaviruses are transmitted between animals and people. Studies on SARS-CoV and MERS-CoV reported that civet cats and dromedary camels were the source for transmission from animal to humans. While n-CoV has been reported to be transmitted through the animal market in Wuhan, China, the epicenter of the outbreak [1].

Signs of n-CoV infection include respiratory symptoms, fever, cough, shortness of breath and breathing difficulties which could worsen to cause pneumonia, severe acute respiratory syndrome, kidney failure and even death especially in elderly.

Standard recommendations to prevent infection spread include regular hand washing, covering mouth and nose when coughing and sneezing and avoiding close contact with anyone showing symptoms of respiratory illness such as coughing and sneezing.

Possible routes of transmission of n-CoV have been identified as airborne, contact spread and spread via contaminated surfaces. Hence, a large number of countries have imposed travel bans, are suspending public gatherings and are screening populations to identify people at risk [2].

Healthcare professionals and staff in hospitals are particularly vulnerable to acquire the infection from the symptomatic as well as asymptomatic individuals due to the aerosols emitted in the air from invasive procedures, proximity to the patients during examinations and contact with contaminated instruments or surfaces [2].

Studies are in progress targeting the characteristics of the virus, factors promoting the spread of the virus, clinical trials to assess the most effective drugs and vaccines, knowledge attitude and practices in populations related to the n-CoV. Recent studies done to assess the awareness about COVID-19 amongst dental workforce focus on awareness about specific practices which help reduce the spread of infection [3–9]. However, we came across no study done as yet to assess whether the dental workforce is aware about the facts and myths related to Covid-19 awareness.

This study aims to analyze and compare the level of awareness about the facts and myths related to COVID 19 amongst faculty, dental students of COD and preparatory year students as part of an awareness campaign.

## Methods

### Test instrument

The covid-19 awareness test conducted at the College of Dentistry was designed using information from the World Health Organization’s (WHO) Myth Busters Awareness webpage (March 2020) [10]. The main purpose of the test was to assess how well the respondents can differentiate between the facts and myths related to the Corona Virus. The online test consisted of 35 MCQ items and was administered to all Faculty, dental students and preparatory year students (prospective students taking admission at the College of Dentistry) using a secure enterprise online assessment platform (Blackboard).

### Research question

What is the true level of awareness amongst faculty, prep year students and undergraduate dental students about the facts and myths related to corona virus?

### Hypothesis

- Faculty are more knowledgeable and can correctly distinguish the facts and myths related to COVID 19.
- There is no difference in knowledge about the facts and myths related to COVID 19 amongst the prep year and dental students.

### Setting

Faculty, dental students of the College of Dentistry and preparatory year students who had applied for the admission to the dental college.

### Study period

The tests were administered over a period of three months from March to June 2020.

### Consent

A Written informed Consent was obtained from all participants in the start regarding the usage of their responses for the publication purpose.

### Ethical information

Ethical Approval was approved by the institutional review board at college of dentistry, IAU holding number EA: 202049.

### Statistical analysis

The data was analyzed using descriptive statistics and summarized by frequency, percentages, mean and standard deviations. One-way ANOVA was used to determine the mean difference among the faculty, dental students and prep year student’s knowledge score. Chi-square test was used to check the associations of myth and facts of the three groups. All the analysis was carried out on software Statistical Package for Social Science (SPSS, version 22 Inc, IBM). All tests were performed at a significance level of α = 0.05.

## Results

The online COVID-19 awareness test was administered to 810 participants, out of which 325 (40%) were prep year students, 429 (53%%) were dental students, and 56 (7%) were faculty members. The average time spent filling the questionnaire was 12.8 mins (range: 1 to 45 mins) by each participant.

Out of 810 participants, 86% of the Faculty were able to correctly identify the facts and the myths related to COVID-19 followed by 81% of the prep year students and 74% of the dental students. Table 1 shows the overall participant’s mean knowledge score out of maximum 35. Preparatory year student’s knowledge related to COVID-19 was found to be high as compared to dental students (26.47±4.247) and most senior dental students of the college also have higher knowledge score of (25.82±4.317).

**Table 1:** Mean knowledge score for faculty members, dental students and preparatory year students.

Participants		N	Minimum	Maximum	Mean	Std. Deviation
Prep Students		325	12	35	26.47	4.278
Dental Students	2nd year dental	32	0	31	21.63	6.617
	3rd year dental	78	0	32	23.04	6.867
	4^th^ year dental	78	0	31	23.91	5.530
	5th year dental	93	0	32	21.91	7.407
	6th year dental	83	3	33	25.82	4.317
	Intern’s	65	0	33	24.94	4.707
Faculty		56	0	33	23.30	7.34

Overall knowledge score comparison was performed to check the differences among the three groups, and it was found significant (P<0.001). Multiple comparison showed that prep year students’ knowledge score was significantly higher than dental student (26.47±4.27, 23.67±6.2 respectively) (P<0.001) and even faculty members (23.30±7.34) (P<0.001). however, the knowledge score of dental students was almost same as faculty members and difference was not statistically significant (p-0.808).

### Fact versus Myth—Comparison among the participants

Table 2 represents the overall item wise comparison of fact and myth between the Faculty; dental students and preparatory year students. All items in related to facts showed the significant proportional difference between the groups except items 10, 11, 12 and 15 showed the same level of knowledge between three groups (faculty, prep year and dental students). All three groups showed almost the same ability in recognizing the myth stating that ‘COVID-19 is old disease and effective vaccine of COVID in available.

**Table 2:** Overall item wise comparison of fact and myth between faculty members, dental students and preparatory year students.

No.	Items	Response	Dental Students	Faculty	Prep	P-Value
OVERALL FACTS						
Q1	Sharing facts helps to reduce fear and stress in yourself and others	Facts	71.0%	84.9%	88.3%	0.0001
		Myths	14.4%	9.4%	8.0%	
Q2	COVID-19 is RNA virus	Facts	70.2%	86.8%	77.8%	0.011
		Myths	18.8%	5.7%	11.4%	
Q3	COVID-19 is transmitted from person to person	Facts	75.5%	92.5%	97.8%	0.0001
		Myths	7.9%	1.9%	1.8%	
Q6	It is possible that a person can get COVID-19 by touching a surface or object	Facts	72.4%	96.2%	81.5%	0.001
		Myths	16.6%	1.9%	7.4%	
Q10	Professional N95 mask can protect health care workers as they care for infected patients	Facts	76.3%	88.7%	83.1%	0.115
		Myths	0.2%	0.0%	0.0%	
Q11	You should cough and sneeze into a tissue, then through it and wash your hands	Facts	94.7%	96.2%	96.9%	0.087
		Myths	2.2%	1.9%	2.8%	
Q12	Disease can make anyone sick regardless of their race or ethnicity	Facts	89.2%	94.3%	85.2%	0.107
		Myths	4.8%	5.7%	8.3%	
Q13	The common symptoms of COVID-19 are fever, cough and shortness breath	Facts	76.1%	96.2%	97.5%	0.001
		Myths	10.9%	1.9%	1.8%	
Q15	COVID-19 seems to be spreading easily and sustainably in the community	Facts	89.9%	92.5%	92.6%	0.551
		Myths	5.3%	3.8%	4.9%	
Q17	Creating stigma because of COVID-19 hurts everyone by creating more fear or anger	Facts	58.8%	69.8%	51.1%	0.04
		Myths	28.1%	22.6%	30.8%	
Q19	You can use diluted bleach solution as a surface disinfectant.	Facts	66.8%	64.2%	42.2%	0.0001
		Myths	15.4%	13.2%	28.3%	
Q21	Thermal scanners are effective in detecting people who have developed a fever	Facts	70.3%	90.6%	79.1%	0.0001
		Myths	21.8%	1.9%	10.5%	
Q22	COVID-19 virus can be transmitted in areas with hot and humid climates	Facts	40.2%	47.2%	40.9%	0.004
		Myths	29.6%	9.4%	21.5%	
Q27	Smoking is ineffective against COVID-19 and can be harmful	Facts	58.1%	75.5%	76.9%	0.001
		Myths	28.9%	7.5%	8.6%	
Q30	Quarantine for 14 days is recommended as this is the longest incubation period seen for similar coronaviruses.	Facts	88.0%	84.9%	86.2%	0.012
		Myths	6.5%	0.0%	8.6%	
Q31	People who are at high risk from COVID-19 infection, includes older adults and pregnant women	Facts	70.7%	88.7%	85.8%	0.001
		Myths	19.7%	0.0%	7.1%	
Q33	Washing your hands frequently is one of the best ways to protect you from infection	Facts	76.9%	98.1%	99.1%	0.001
		Myths	15.2%	1.9%	.3%	
Q34	You should wear a mask if you are caring for someone who is sick	Facts	89.7%	92.5%	96.0%	0.002
		Myths	5.3%	1.9%	3.7%	
OVERALL MYTHS						
Q4	COVID-19 is an old disease, caused by coronavirus that has previously been seen in humans.	Facts	36.7%	22.6%	39.1%	0.252
		Myths	48.8%	58.5%	47.1%	
Q5	COVID-19 is the same as SARS	Facts	39.6%	24.5%	18.5%	0.0001
		Myths	38.2%	45.3%	57.8%	
Q7	An effective vaccine to cure COVID-19 is available	Facts	2.6%	1.9%	1.8%	0.868
		Myths	55.4%	50.9%	53.5%	
Q8	You can protect yourself from infection by gargling bleach or using essential oils	Facts	25.3%	5.7%	6.8%	0.001
		Myths	59.0%	81.1%	65.2%	
Q9	Wearing a face mask generally is recommended to protect you from COVID-19.	Facts	59.5%	54.7%	96.9%	0.001
		Myths	28.3%	22.6%	2.5%	
Q14	Children below 11 years old are safe from the virus	Facts	21.2%	7.5%	2.5%	0.0001
		Myths	62.5%	60.4%	75.7%	
Q16	Hand dryers are effective in killing COVID-19	Facts	24.6%	5.7%	13.5%	0.0001
		Myths	54.0%	62.3%	58.2%	
Q18	Cold weather can kill COVID-19 virus	Facts	8.4%	0.0%	2.2%	0.001
		Myths	64.0%	81.1%	65.5%	
Q20	Antibiotics are effective in preventing and treating COVID-19 infection	Facts	5.1%	0.0%	9.8%	0.001
		Myths	54.9%	66.7%	56.3%	
Q23	Ultraviolet disinfection lamp can kill COVID-19 virus	Facts	31.3%	17.0%	5.5%	0.0001
		Myths	28.9%	26.4%	37.2%	
Q24	Spraying alcohol or chlorine all over your body can kill COVID-19 virus	Facts	37.3%	18.9%	23.7%	0.0001
		Myths	48.9%	62.3%	53.8%	
Q25	There are specific validated medicines to treat the new coronavirus	Facts	29.9%	3.8%	8.3%	0.001
		Myths	23.7%	30.2%	37.5%	
26	Eating garlic can help to prevent COVID-19 infection	Facts	24.5%	3.8%	7.1%	0.001
		Myths	56.8%	67.9%	52.9%	
28	Regularly rinsing your nose with saline prevents infection with COVID-19	Facts	27.8%	18.9%	15.1%	0.001
		Myths	44.4%	41.5%	44.3%	
Q29	Taking a hot bath prevents COVID-19 disease	Facts	4.6%	5.7%	1.8%	0.001
		Myths	76.0%	73.6%	64.9%	
Q35	Vaccine against pneumonia protects from COVID-19 infection	Facts	13.2%	0.0%	7.4%	0.001
		Myths	39.4%	50.9%	33.5%	

Overall, the knowledge of faculty about the facts is higher than the undergraduate students. Faculty was more likely to be aware about the facts than students, but the difference is not significant (86%, 74%, respectively).

**Fig 1:**
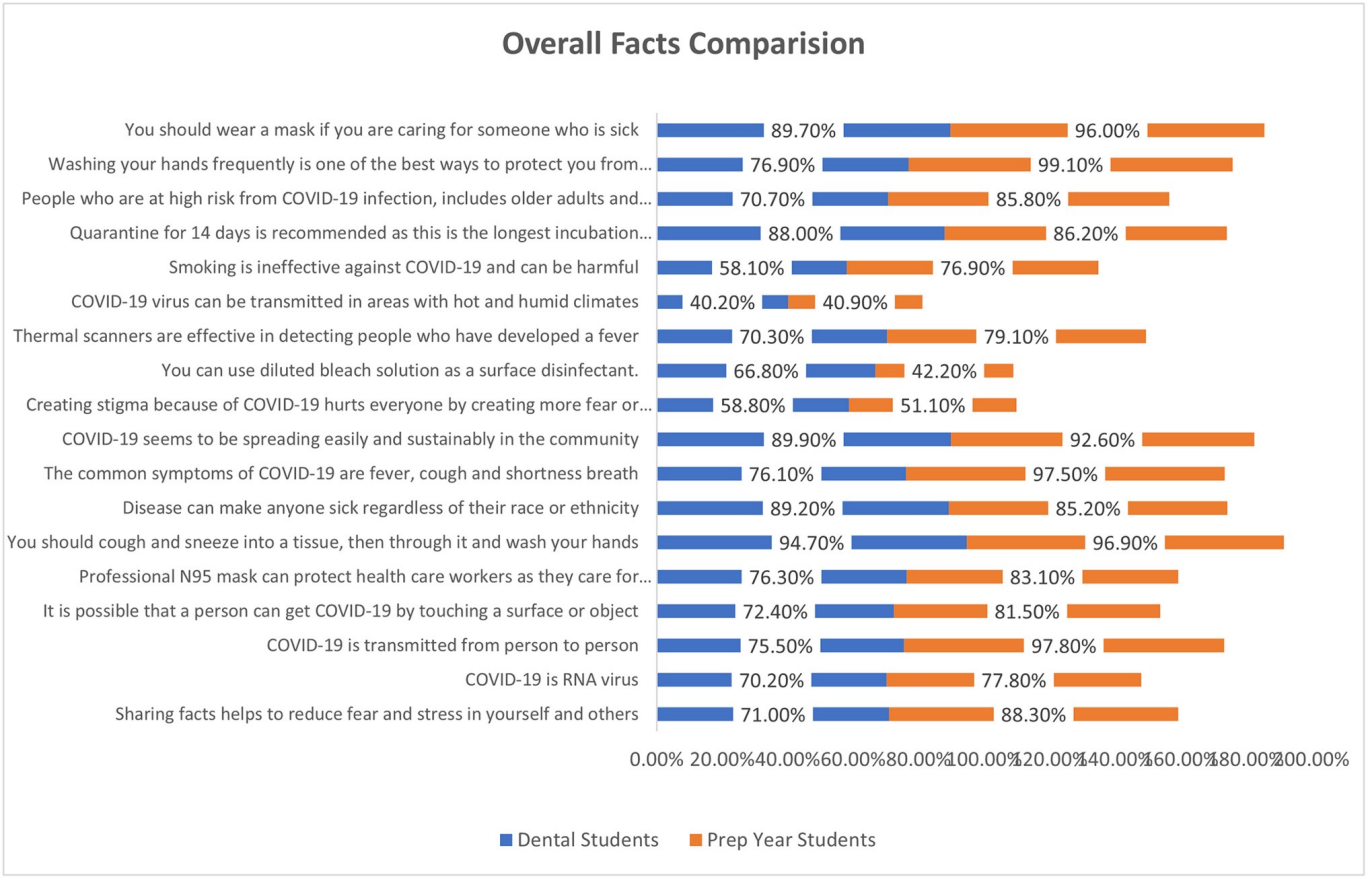
Overall facts comparison between dental and prep year students.

**Fig 2:**
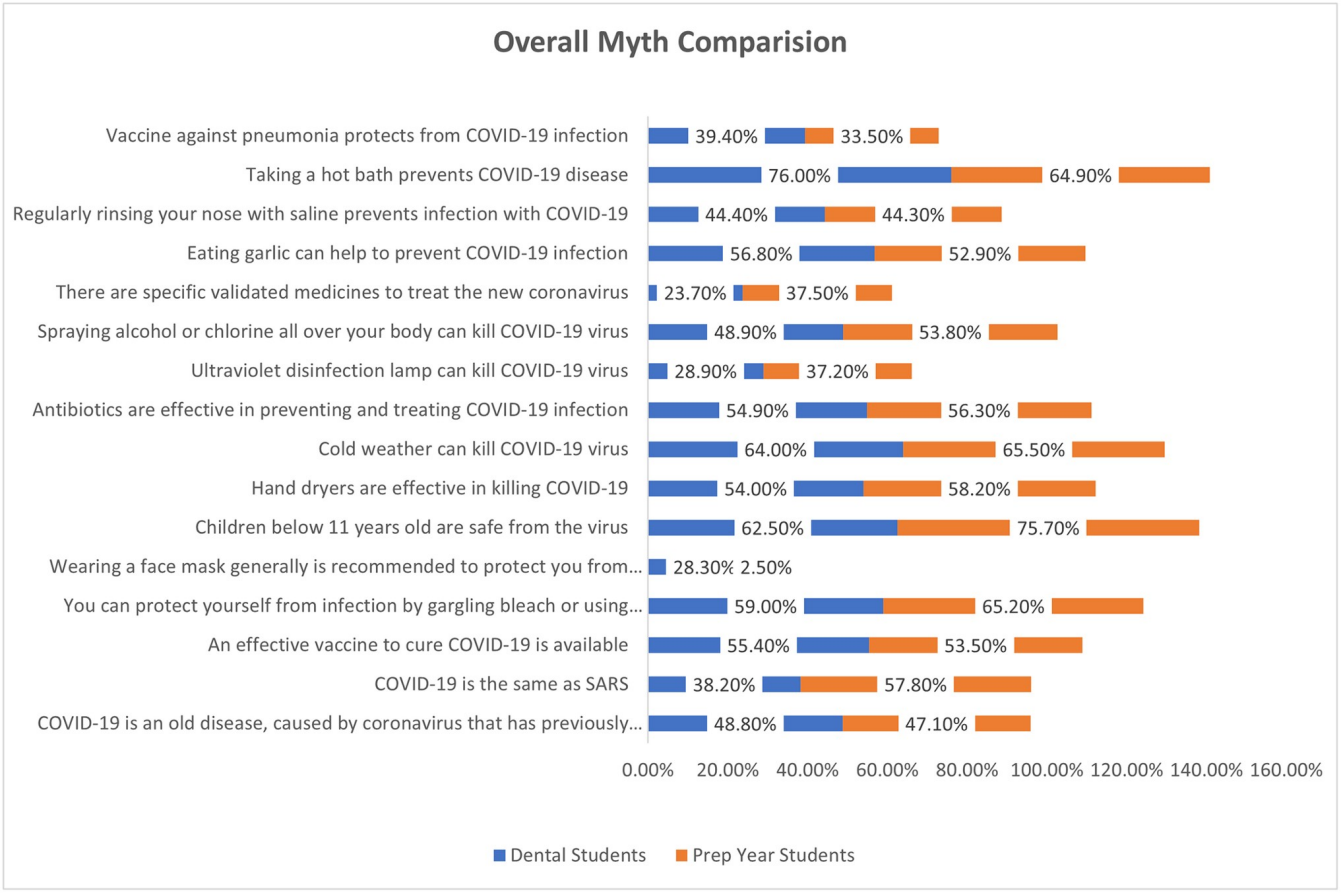
Overall Myths comparison between dental and prep year students.

Figs 1 and 2 showing the overall facts and myths comparison between dental and prep years students. Dental student’s awareness to the facts was little lower as compared to the prep year students whereas the difference was negligible about the myths between both groups.

### Myths that were considered facts and vice versa by dental students

Table 3 presents the list of top five Myths that were considered as facts by the dental students. Thirty one percent (31%) of the dental students thought that ultraviolet light can help to kill COVID-19 virus, this was significantly higher as compared to the faculty among whom only 17% considered it as a fact. Wearing a mask generally is recommended to protect you from COVID-19 was considered as fact by majority of dental students (59%). Whereas around 30% dental students wrongly marked the facts that COVID-19 virus can be transmitted in hot and humidity and smoking is ineffective against COVID-19 (29%), as myths and these proportions were significantly different than faculty’s response.

**Table 3:** Top five Myths that were considered facts and vice versa by dental students.

**Myths that were considered facts**		
Question No.	Questions	Percentage of Dental students who considered myth as fact
9	Wearing a face mask generally is recommended to protect you from COVID-19.	59.5%
5	COVID-19 is the same as SARS	39.6%
24	Spraying alcohol or chlorine all over your body can kill COVID-19 virus	37.3%*
4	COVID-19 is an old disease, caused by coronavirus that has previously been seen in humans.	36.7%
23	Ultraviolet disinfection lamp can kill COVID-19 virus	31.3%*
**Facts that were considered Myths**		
Question No.	Questions	Percentage of Dental students who considered fact as myth
22	COVID-19 virus can be transmitted in areas with hot and humid climates	29.6%*
27	Smoking is ineffective against COVID-19 and can be harmful	28.9%*
17	Creating stigma because of COVID-19 hurts everyone by creating more fear or anger	28.1%
21	Thermal scanners are effective in detecting people who have developed a fever	21.8%*
31	People who are at high risk from COVID-19 infection, includes older adults and pregnant women	19.7%*

* significant at 0.05 in comparison with the faculty knowledge.

### Myths that were considered facts and vice versa by faculty members

Similarly, like dental students’ majority of the faculty 55% incorrectly marked that wearing a mask is recommended to protect against COVID-19. Nineteen percent (19%) of them thought the fact spraying alcohol chlorine can kill the virus is a myth, but this percentage was significantly lesser than dental students among whom 37% considered it a myth.

Creating stigma because of COVID-19 hurts everyone more this fact was considered as myth by 23% of the faculty and using diluted bleach as surface disinfection was also marked wrongly by the 13% faculty. Only two items were marked significantly lesser than dental students were 22 and 27 see Table 4.

**Table 4:** Top five Myths that were considered facts and vice versa by faculty members.

**Myths that were considered facts**		
Question No.	Questions	Percentage of faculty who considered myth as fact
9	Wearing a face mask generally is recommended to protect you from COVID-19.	54.7%
5	COVID-19 is the same as SARS	24.5%
4	COVID-19 is an old disease, caused by coronavirus that has previously been seen in humans.	22.6%
24	Spraying alcohol or chlorine all over your body can kill COVID-19 virus	18.9%*
28	Regularly rinsing your nose with saline prevents infection with COVID-19	18.9%
**Facts that were considered Myths**		
Question No.	Questions	Percentage of faculty who considered fact as Myth
17	Creating stigma because of COVID-19 hurts everyone by creating more fear or anger	22.6%
19	You can use diluted bleach solution as a surface disinfectant.	13.2%
1	Sharing facts helps to reduce fear and stress in yourself and others	9.4%
22	COVID-19 virus can be transmitted in areas with hot and humid climates	9.4%*
27	Smoking is ineffective against COVID-19 and can be harmful	7.5%*

*Significant at 0.05 in comparison with Dental Students.

### Myths that were considered facts and vice versa by prep year students

The myth that COVID-19 is an old disease cause by an old coronavirus was incorrectly recognized by 40% preparatory year students as a fact (Table 5). Another large number of preparatory year student (24%) thought spraying alcohol or chlorine on your body can kill virus but this number was still significantly less than dental students (34%). Another big difference was observed about myth that was considered as fact by preparatory year and dental students (14%, 25% respectively) that hand dryers are effective in killing virus. Top facts which were marked as myth by the preparatory year students were “Creating stigma because of COVID-19 hurts everyone (31% considered it a myth)”, diluted “bleach as surface disinfector (28% considered it a myth)” and “COVID-19 virus can be transmitted in hot and humidity area 22% considered it a myth” and these incorrect responses were significantly different from the responses of faculty and dental students.

**Table 5:** Top five Myths that were considered facts and vice versa by preparatory year students.

**Myths that were considered facts**		
Question No.	Questions	Percentage of Prep students who considered myth as fact
4	COVID-19 is an old disease, caused by coronavirus that has previously been seen in humans.	39.1%
24	Spraying alcohol or chlorine all over your body can kill COVID-19 virus	23.7%*
5	COVID-19 is the same as SARS	18.5%*
28	Regularly rinsing your nose with saline prevents infection with COVID-19	15.1%*
16	Hand dryers are effective in killing COVID-19	13.5%*
**Facts that were considered Myths**		
Question No.	Questions	Percentage of Prep students who considered myth as fact
17	Creating stigma because of COVID-19 hurts everyone by creating more fear or anger	30.8% ^β^
19	You can use diluted bleach solution as a surface disinfectant.	28.3%* ^β^
22	COVID-19 virus can be transmitted in areas with hot and humid climates	21.5%*^β^
2	COVID-19 is RNA virus	11.4%*
21	Thermal scanners are effective in detecting people who have developed a fever	10.5%*

*significant at 0.05 in comparison with Dental Students.

^β^ significant at 0.05 in comparison with Faculty.

## Discussion

Very few studies have tried to assess whether the healthcare workforce is clear and vigilant about the facts and myths related to the corona virus. Most of the studies focus on the knowledge, attitude and practice related to awareness about specific practices in reducing the spread of infection [3–9].

It is imperative for healthcare professionals to be able to discern between the facts and myths related to the transmission of corona virus. Since things are not black and white or “clear cut” when dealing with a new infectious disease, it becomes the responsibility of the healthcare provider to follow the facts and be the myth busters.

This study used the “COVID-19 Advice for Public: MythBusters” shared by WHO [10] to prepare the 35 items test to assess whether the faculty and students at the College of Dentistry could correctly differentiate between the facts and myths related to COVID -19, as part of the awareness campaign.

Results of the test showed that 86% of the faculty, 74% dental students and 81% of the prep year students were able to correctly differentiate between the facts and the myths. Faculty members were the first group to be administered the test and the preparatory year students the last group during the three-month study period from April to June. The reason for the low percentage of correct answers from the faculty and high percentage of correct answers from the preparatory year students could be because of the timing of the tests where the preparatory year students benefitted from the updated information shared on a regular basis by the Ministry of Health and WHO leading to increased awareness towards the end of the study period.

Generally, there was significantly higher awareness about the facts related to the COVID-19 symptoms, spread of the disease, use of face mask and appropriate quarantine by the majority of the respondents in all groups. These findings concur with other studies focusing on knowledge, attitudes and practices related to COVID-19 pandemic [3–9].

The results showed that facts were easier than myths to be perceived among different groups as more than 70% of participants answered the facts correctly. On the other hand, most myths were challenging, especially those related to prevention and treatment.

“Wearing a face mask generally is recommended to protect you from COVID-19” was a Myth according to the WHO Myth Busters webpage. In our study, this myth was one of the top 5 myths considered as a fact by 60% of the dental students and 55% of the faculty. WHO website is also updating the MythBusters regularly and this particular myth has now been updated as a “Fact” with additional information regarding wearing a mask during exercise and for prolonged time, also the difference between various types of available masks. Recent study highlights the importance of use of face masks with social distancing of 1 m or more as a potential barrier against the rapid spread of COVID-19 infection [11–13].

Outbreak of COVID-19 pandemic led to increased social stigmatization at various levels, from those who got infected and recovered to the Chinese community from where the disease is believed to have spread [14]. The statement that “Creating stigma because of COVID-19 hurts everyone by creating more fear or anger” was a “Fact” according to the WHO Myth Buster webpage, however it was wrongly identified as one of the top five “Myths” by 31% of the preparatory year students, 28% of the dental students and 22% of the faculty members. As part of the healthcare workforce, increased awareness should be directed towards the negative side of creating stigma [14], emphasizing that 96% of people have recovered till date from the COVID-19 infection [15] and that this is not a life-long disease like Acquired Immunodeficiency Disease (AIDS). WHO has also recently introduced a portal for reporting any misleading information related to COVID-19 as part of its awareness campaign to minimize spread of false or misleading information related to COVID-19 [16].

Similarly, the statement “COVID-19 is the same as SARS” identified as a “Myth” according to the WHO Myth Buster webpage was wrongly identified as a “Fact” by 40% of the dental students; 25% of the faculty members and 19% of the preparatory year students.

Another common myth that there are specific validated medicines for treatment, was found to be prevalent among the dental students (55%%) and faculty (51%) followed by preparatory year students (53%). This brings us to the importance of awareness about the process for validating medicines. Health care workers should be aware that there are several ongoing drug trials and that there is currently no proof that hydroxychloroquine or any other drug can cure or prevent COVID-19. Misuse of medicine can cause serious side effects and illness and even lead to death [17]. However, those infected with the virus should receive appropriate care to relieve and treat symptoms, and those with severe illness should receive optimized supportive care.

The myth that pneumococcal vaccine can be used to provide protection against COVID-19 was found to be prevalent among the dental students and preparatory year students (39%, 34% respectively) with 51% of the faculty also believing it while the fact is that there is no validated vaccine for COVID-19 till now.

## Conclusion

This study reports about a successful pilot test conducted to assess the perceived knowledge about facts and myths related to corona virus amongst the dental workforce. Results from the study guide the administrators to focus on the facts in the multiple awareness sessions and processes and emphasizes the role of the healthcare provider as myth-busters.

## Recommendations

Information should be acquired from International organizations, such as WHO, CDC or Government Health Ministry instead of following the news spread through multiple news channels or social media.

Regular awareness campaigns should be conducted and followed by pre and post test of knowledge attitude and practices, Signage and posters related to important facts and the busting of the most prevalent myths should be put up in all public places, workplaces, and institutions as a constant reminder.
